# Development of a hybrid point-of-care ultrasound curriculum for first year medical students in a rural medical education program: a pilot study

**DOI:** 10.1186/s12909-023-05005-6

**Published:** 2024-01-03

**Authors:** Joshua I. Johnson, Heather Beasley, Derek Southwick, Allie M. Lords, Ross Kessler, Michael E. Vrablik, Russell T. Baker

**Affiliations:** 1 WWAMI Medical Education Program, University of Idaho, Moscow, Idaho USA; 2 University of Washington School of Medicine, Seattle, Washington USA; 3 Department of Emergency Medicine, University of Washington, Seattle, Washington USA

**Keywords:** Curriculum development, Point of care ultrasound, Interprofessional education, Clinical skills, Anatomical sciences

## Abstract

**Background:**

The field of point-of-care ultrasound (POCUS) has advanced in recent decades due to the benefits it holds for medical providers. However, aspiring POCUS practitioners require adequate training. Unfortunately, there remains a paucity of resources to deliver this training, particularly in rural and underserved areas. Despite these barriers, calls for POCUS training in undergraduate medical education are growing, and many medical schools now deliver some form of POCUS education. Our program lacked POCUS training; therefore, we developed and implemented a POCUS curriculum for our first-year medical students.

**Methods:**

We developed a POCUS curriculum for first year medical students in a rural medically underserved region of the United States. To evaluate our course, we measured learning outcomes, self-reported confidence in a variety of POCUS domains, and gathered feedback on the course with a multi-modal approach: an original written pre- and post-test, survey tool, and semi-structured interview protocol, respectively.

**Results:**

Student (*n*=24) knowledge of POCUS significantly increased (pre-test average score = 55%, post-test average score = 79%, *P*<0.0001), and the course was well received based on student survey and interview feedback. In addition, students reported increased confidence toward a variety of knowledge and proficiency domains in POCUS use and their future clinical education and practice.

**Conclusions:**

Despite a lack of consensus in POCUS education, existing literature describes many curricular designs across institutions. We leveraged a combination of student initiatives, online resources, remote collaborations, local volunteers, and faculty development to bring POCUS to our institution in a rural and medically underserved region. Moreover, we demonstrate positive learning and experiential outcomes that may translate to improved outcomes in students’ clinical education and practice. Further research is needed to evaluate the psychomotor skills, broader learning outcomes, and clinical performance of students who take part in our POCUS course.

## Introduction

### Ultrasound in medical practice and education

Ultrasound has been integrated at the point of care in many specialties to answer clinical questions at the bedside [1]. This is often termed point-of-care ultrasound (POCUS), or sonology [2]. The inception and expansion of POCUS in clinical practice is driven by several factors. Technological advancements have reduced equipment cost, size, and complexity, while increasing usability, imaging quality, and portability [2–5]. Further, POCUS can safely generate reliable and efficient (e.g., time, cost) real time images that provide valuable clinical information to guide medical decisions [1, 4–9].

Thus, POCUS is a powerful clinical tool with numerous and significant benefits; however, there are challenges and barriers to clinical implementation. For example, POCUS users must acquire, optimize, and interpret the requisite sonogram(s) for a given clinical scenario. Therefore, aspiring sonologists must develop a unique blend of competency and proficiency in appropriate cognitive domains and psychomotor skills [2, 10, 11]. Consequently, effective POCUS users require training that currently lacks formal standards in undergraduate medical education (UME).

Despite the lack of uniform requirements, calls from professional bodies [7, 12, 13], students [14–16], and expert panels [17] have increased students’ exposure to POCUS, and curricula are now incorporated at many American medical schools [18]. However, in the absence of standards, the existing US curricula are heterogeneous [18], ranging from short workshops [16] or self-led online learning [19], to longitudinal four-year curricula [7, 14]. Therefore, decisions on curricular content and organization remain an important concern for schools wishing to implement new POCUS courses [20]. Moreover, UME programs located in rural or underserved areas such as ours face additional challenges [21, 22].

### Rural and urban divides in ultrasound

American medical schools are concentrated in urban areas where they benefit from a relative abundance of medical resources [23, 24]. By contrast, UME institutions in less populated areas, particularly in the Northwest, are disproportionately affected by the scarcity of medical services [25]. While barriers to POCUS teaching such as such as a shortage of equipment, faculty, and an overburdened curriculum are well-documented across many institutions [7, 18, 26–32], these challenges are heightened in rural and medically underserved regions. For instance, recent data indicates that just 39% of rural counties in the United States had access to POCUS, compared to 89% of metropolitan counties [22]. This disparity impacts public access to healthcare and presents challenges for medical educators aiming to implement POCUS training in these communities [21].

While rural POCUS education initiatives have been detailed, many describe outreach programs developed in highly resourced institutions targeting practitioners in low- and middle-income countries [21, 26–29]. By contrast, descriptions of POCUS teaching developed within rural American UME programs are underrepresented in the literature. Our institution, the University of Idaho, functions as a regional campus in the WWAMI (Washington, Wyoming, Alaska, Montana, Idaho) medical education program [25]. Idaho ranks among the most rural and medically underserved states [30–32], and, more broadly, the Northwest region of the country has suffered a persistent lack of medical resources for decades [23, 25]. Moreover, the clerkship years of our curriculum are distributed across clinical training sites throughout the five-state WWAMI region, decentralizing clinical resources (e.g., clerkship faculty, dedicated clinical teaching facilities) that are often used to facilitate POCUS teaching during the pre-clinical phase on other campuses.

In 2021, an Ultrasound Student Interest Group (USIG) chapter was established with the incoming cohort of students at the Idaho WWAMI campus. In response to the lack of local ultrasound education, USIG leadership consolidated a patchwork of asynchronous, extracurricular, and USIG-generated POCUS resources from other regional WWAMI sites, developed a list of learning objectives desired to be formalize in the basic science curriculum, and petitioned for institutional support (e.g., faculty, administrative, and budgetary resources) to do so. The efforts resulted in a collaborative taskforce that worked to develop a curriculum including didactic and hands-on POCUS training for the preclinical medical students on our regional campus, which followed a six-step procedure outlined by Thomas and colleagues [33].

The current paper contributes to the literature by describing the implementation of a hybrid POCUS curriculum novel to our rural and underserved regional setting. Our discussion covers curriculum development, knowledge outcomes, and student experiences in the inaugural POCUS course for first year medical students (MS1). Contributing to the broader conversation on integrating POCUS into health professions education, we emphasize strategies tailored to the specific challenges faced by institutions in rural and underserved regions.

## Methods

To generate a POCUS curriculum in our local context, we first performed an informal survey of relevant literature. Our search revealed an extensive body of literature describing the increasing clinical and academic integration of POCUS [4, 5, 8, 12–14, 16, 18, 20, 34–39]. Moreover, national surveys [18], consensus statements, recommendations from field experts [12, 13, 40], and published POCUS guides [1, 41, 42] further enriched our understanding of the POCUS landscape in practice and UME.

While these sources provided valuable insights, our lack of institutional knowledge in the clinical and didactic use of POCUS prompted us to seek further input. We collaborated with clinical faculty (e.g., MD/DO Emergency Physicians with fellowship training in POCUS, and experience with POCUS curriculum development) at the University of Washington School of Medicine (UWSOM) – the parent institution for the WWAMI program – and reached out to extramural clinical faculty and practitioners for further discussion and refinement of the USIG proposed learning objectives.

The collaborative process yielded a set of overarching goals, identified barriers specific to our local context, and outlined feasible strategies to overcome these challenges. A summary of these findings is presented in Table 1. Moreover, we refined the curriculum and learning objectives proposed by the USIG and organized a series of guest lecturers to deliver content and support scanning instruction. These are summarized in Table 2.

**Table 1:** Local goals, barriers, and strategies around POCUS training

**Goals for the curriculum**	1. Develop students’ knowledge of the principles of POCUS, and clinical indications for its use. 2. Develop students’ proficiency in image acquisition and interpretation. 3. Promote student engagement and learning in the basic science curriculum with POCUS.
**Local Barriers**	Our regional campus hosts an 18-month basic science curriculum resulting in: • Limited time frame for implementing additional coursework. • Limited access to advanced students, clinical faculty, and teaching resources In addition, we identified contextual barriers including: • Minimal institutional knowledge in POCUS. • No POCUS equipment for hands-on training. • Limited community medical resources (e.g., critical access hospitals). • Many competing demands on student and institutional resources.
**Local Strategies**	To bring POCUS training to our campus, we identified the following strategies: • Leverage appropriate FOAMed and asynchronous resources. • Interdepartmental purchase of POCUS equipment to defray costs. • Integration of POCUS content after relevant teaching in the basic science curriculum. • Bolster intramural faculty with: ○ Faculty development initiatives ○ Local registered diagnostic medical sonographers ○ Local physician POCUS users ○ Remote physician POCUS users

Summarized goals, local barriers, and mitigating strategies

Acronyms: *FOAMed* Free Open Access Medical education, *POCUS* Point of Care Ultrasound

**Table 2:** Course topics, learning objectives, and faculty characteristics

**POCUS Topic**	**POCUS Learning objective:** **Following the completion of this session, successful students will be able to:**			
**Introduction to Ultrasound:**	1. Describe the basic physics of image transduction in B-Mode sonography including definitions of ultrasound specific terms such as echogenicity and attenuation. 2. Differentiate various tissue types in the neck based on their echotexture. 3. Correlate sonographic images with probe placement, surface, gross, and cross-sectional anatomy, to identify various anatomical structures and landmarks. 4. Practice probe handling techniques to thoroughly examine structures in long and short axis. 5. Define linear, curvilinear, and phased array probe types in terms of their operating frequency, imaging characteristics, and suitable applications. 6. Recognize and describe the physical mechanisms of common imaging artifacts, including reverberation artifact, attenuation artifacts, edge artifact, and scatter artifact. 7. Demonstrate understanding of color doppler by employing it to differentiating neurovascular structures. 8. Identify the leading-edge, receding edge, near field, and far field of sonograms, and orient the anatomical directions (e.g., anterior, posterior, superficial, deep, superior, inferior) of an image based on the orientation of the probe and probe marker. 9. Capture and submit an image clip to show proficiency in use of the Butterfly ultrasound technology.			
**Common to all other topics:**	1. List the common clinical indications for performing POCUS studies in this system/organ/region. 2. Execute the appropriate procedures for image acquisition, including equipment/preset selection, probe orientation, patient position, image optimization, and navigating relevant pearls and pitfalls. 3. Recognize the sonographic features of normal anatomy in this system/organ/region. 4. Describe the sonographic findings associated with common pathological or abnormal findings underlying common clinical indications relevant to the organ system or region of interest.			
	**Faculty Characteristics**			
	**Specialty**	**Credential**	**POCUS Training**	**Location**
**1. Intro to ultrasound**	Emergency Medicine	DO	Fellowship	Remote, UWSOM
**2. Cardiac POCUS**	Emergency Medicine	MD	Residency	Remote, Extramural
**3. Lung POCUS**	Internal Medicine	MD, FACP, RDMS	RDMS	Local, Extramural
**4. Aorta and IVC**	Emergency Medicine	DO	Fellowship	Remote, UWSOM
**5. POCUS-Guided Vascular access**	Emergency Medicine	MD	Residency	Local, Intramural (part-time)
**6. Renal/Urinary system**	Anatomist	MS	Faculty Development	Local, Intramural
**7. Obstetrics**	Internal Medicine	MD, FACP, RDMS	RDMS	Local, Extramural
**8. eFAST**	Family Medicine	MD	Residency	Remote^a^, Extramural

A summary of learning objectives for the course, formulated along the International Training and Education Center for Health (I-TECH) guidelines for writing good objectives [44]. Faculty Characteristics describes the session leader for each topic, responsible for delivering didactic content

Acronyms: *POCUS* Point-of-care ultrasound, *IVC* Inferior vena cava, *eFAST*: Extended focused assessment with sonography in trauma, *MD* Medical Doctor, *DO* Doctor of Osteopathy, *MS* Master of Science, *FACP* Fellow of the American College of Physicians, *RDMS* Registered diagnostic medical sonographer, *UWSOM* University of Washington School of Medicine

^a^This provider was remote but elected to travel from a neighboring community to teach in person

### Course description

Developing a new course required adequate administrative support and coordination. Structuring the project as an elective course provided feasibility (e.g., low administrative effort) and flexibility (e.g., scheduling, content, grading, rigor) in implementing a local curricular intervention. We reviewed our basic science curriculum to identify a suitable location to incorporate POCUS training while considering the competing demands on students, faculty, and teaching spaces. In addition, we worked to minimize additional academic commitment for our students by capitalizing on existing didactic content delivery.

The first semester of our MS1 curriculum contained an introduction to imaging modalities and basic physical exam skills, while the second semester integrated content threads such as gross anatomy, (patho)physiology, pharmacology, etc., in a systems-based approach to the thoracic and abdominopelvic regions. To capitalize on this content, we integrated our elective throughout the second semester.

The final POCUS curriculum incorporated eight 90-minute teaching sessions (Table 2) between January and June of 2022. An introductory session highlighted the principles and instrumentation of sonography essential to the operation and implementation of POCUS [41–43], and subsequent meetings described the applications of POCUS using regional, organ-system, or procedure (e.g., POCUS guided vascular access) focused content. The order of POCUS topics (Table 2) in our curriculum followed the organization of our basic science coursework. Students were evaluated on a pass/fail basis for this pilot: passing required in-class attendance of at least 80%; we tracked attendance through sonograms submitted by students at each session.

### Implementation

Implementing the course required adequate teaching materials, additional faculty time, and budgetary support. We worked with our institution’s directors of curriculum and medical research to allocate funding towards purchase of POCUS equipment, compensation for additional faculty commitment, and honoraria for guest lecturers and scanning guides.

#### Didactic teaching materials

**Fig. 1:**
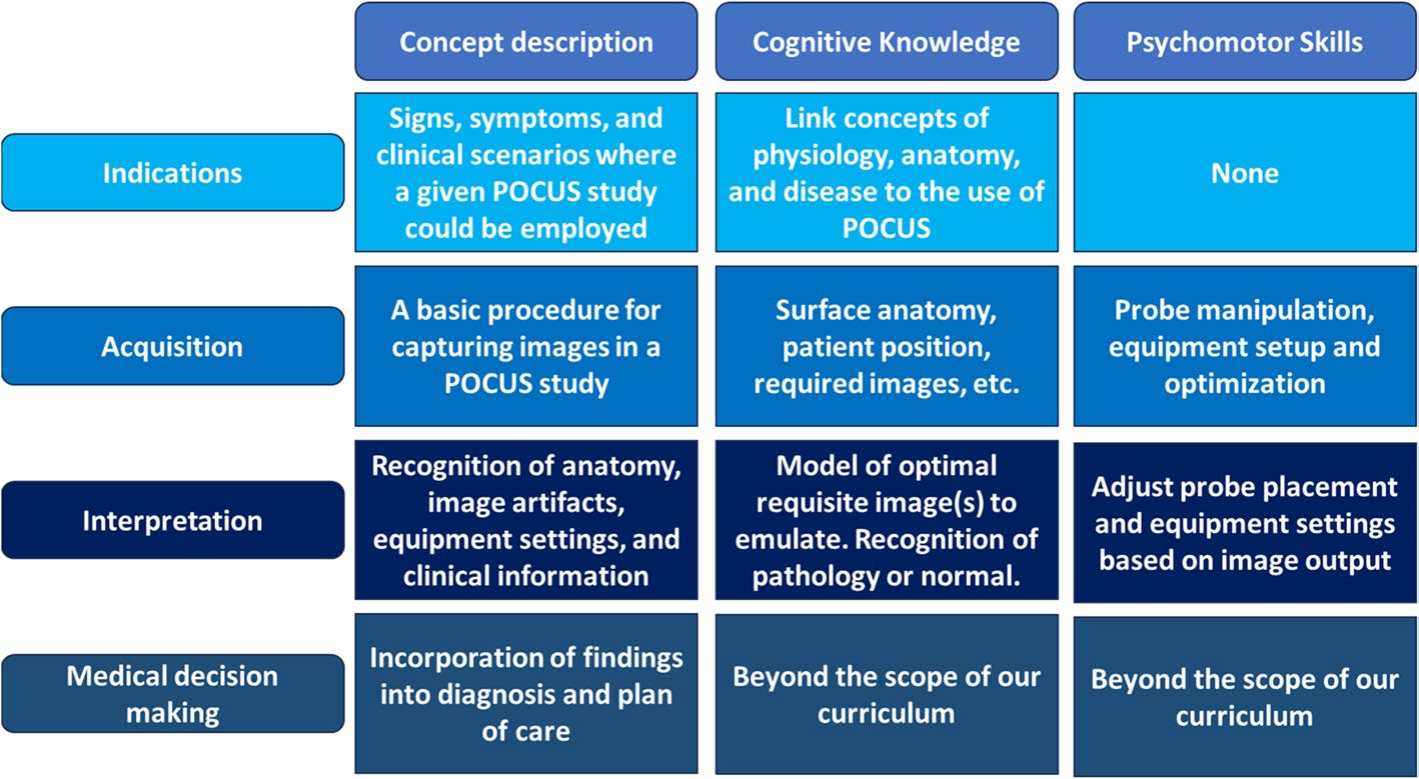
Schematic of the I-AIM framework. This schematic details our operationalization of the four domains in the I-AIM framework for teaching and performing US: Indications, Acquisition, Interpretation, and Medical Decision Making. Each domain is described and broken down into its constituent cognitive and psychomotor domains. It is important to note that acquisition of optimal images is not possible without an active cycle of interpretation and adjustment of the probe and settings. Thus, these two steps are interdependent, and interpretation for clinical findings occurs after image optimization. Finally, we did not include a formal discussion of the medical decision making that follows interpretation of clinical findings in POCUS studies, as this is beyond the scope of our introductory curriculum

Proficiency in image acquisition is predicated on a didactic foundation in the principles of sonography followed by adequate guided scanning practice [10, 12]. Development of course materials was a time intensive process, and we lacked local content experts with sufficient availability. As a result, didactic material tailored to our learning objectives was developed by a POCUS-naïve anatomist as a faculty development project. Resources were adapted from published POCUS guidelines [1] and free open access medical education (FOAMed) resources (e.g., POCUS 101, The POCUS Atlas, CORE Ultrasound) and the indications, acquisition, interpretation, medical decision making (I-AIM; Figure 1) model of ultrasound [45] provided a consistent organizing principle. Materials were reviewed by POCUS-trained collaborators to ensure accuracy. Importantly, we did not include formal discussion of medical decision making as this was more appropriate for clinical settings such as clerkship, residency, or fellowship training [40].

#### POCUS equipment and scanning proficiency

Developing proficiency in image acquisition required hands on training with equipment that our campus lacked. We found a wide variety of available POCUS equipment with diverse capabilities [46]. To guide our purchase decisions, we consulted local sonographers as well as collaborators teaching POCUS in UME, weighed their recommendations, and ultimately acquired 12 Butterfly IQ+ probes (Butterfly Inc., Burlington, MA) due to their combination of affordability, durability, and versatility, including built in education, Teleguidance, and image database features. Equipment costs were defrayed though a joint purchase with our Athletic Training program with plans to expand POCUS curricula across departments and increase return on investment.

A recent proposal suggested an instructor/student ratio of 1:4 to support learning during hands on scanning instruction [12]. While our rural program suffered from a lack of trained sonologists to support student skill development, a variety of suitable scanning instructors have been employed in various POCUS courses, including sonographers [37, 47, 48], anatomists [49, 50], near-peer student teachers [20, 51], self-led instruction [19], and physicians of various specialties [18]. Moreover, remote scanning guidance, such as the Butterfly IQ+ Teleguidance feature, emerged as a novel method to connect students to faculty support [52]. We leveraged all the above categories, resulting in an instructor to student group ratio of 1:2 to 1:4 across sessions, based on volunteer availability.

Students served as both sonologist and scanning models for one hour of hands-on small group scanning practice at each course meeting. For the obstetrics session, we recruited pregnant volunteers from the community to serve as scanning models. In the case of incidental findings in scanning models, our policy was to advise a follow up with their primary care provider; however, no abnormal findings were encountered. In total, our course offered four hours of synchronous didactic input and 12.5 hours of hands-on practice guided by an interprofessional team of faculty, physicians, and sonographers. Finally, we offered three optional scanning practice sessions and developed a procedure for students to check out equipment for self-directed practice [12], though we did not track or quantify the effects of these activities.

### Course evaluation

Course evaluation is an integral part of curriculum development projects [33]. We designed and conducted a prospective observational study to collect outcome data from our inaugural course. This study was certified exempt by the University of Idaho institutional review board, protocol 21-246.

### Participants

First year medical students who chose to enroll in the POCUS elective were given the opportunity to participate in the research component of the course; participation was voluntary and did not affect course standing. Twenty-five of 40 students in the MS1 cohort enrolled in the POCUS elective.

### Informed consent

We collected informed consent separately for the pre/post course knowledge test, post course survey, and semi-structured interviews, respectively. The knowledge tests were prefaced with a statement of informed consent on the Canvas Learning Management System (Instructure, Inc., Salt Lake City, UT). A statement of consent prefaced the post course surveys, which were distributed online via Qualtrics (Qualtrics, Provo, UT; Version 062022). Finally, we collected informed consent from students who participated in face-to-face semi-structured interviews prior to initiating the interview.

### Knowledge test development

Pre/Post tests are a basic and essential component for the evaluation of training programs [53]. Therefore, we developed an original 30-item test consisting of 24 multiple choice (MCQ), three fill in the blank, and three true/false questions. One fill in the blank was a four-part question, bringing the point value of the test to 33. Questions were written to evaluate knowledge in each of the main content areas of the course, including a basic understanding of POCUS technology, image acquisition, and normal image interpretation across a range of organ systems and POCUS applications (Table 2). Multiple faculty collaborators (JIJ, HB, RK, MEV) wrote questions and reviewed the test for accuracy, readability, and difficulty. Comments on initial drafts were assembled and addressed by JIJ prior to administering the exam. Pre-course and post-course tests were administered on Canvas prior to the first class meeting and following the conclusion of the final class meeting, respectively. The content of the pre and post tests were identical. However, to limit the effect of memorizing the responses, we randomized the order of distractors in the MCQs [53].

### Survey development

Proponents in the literature claim that POCUS is a valuable tool for teaching gross anatomy and clinical skills in UME, students trained in POCUS will have greater diagnostic accuracy and improved patient outcomes, and that students are motivated and grateful for POCUS training [3, 15, 20, 38, 54]; we evaluated these themes following course participation. The Preparedness for Hospital Practice Questionnaire [55] is a survey instrument validated to measure various impacts of medical education. We modified this survey by tailoring questions to target the impact of our POCUS training on participants. The survey was modified by JIJ, then reviewed by HB to ensure item relevance and appropriateness. Finally, RTB reviewed the items, as well as overall survey design and format.

Likert questions included 6-point response scales across four domains (Table 3). Additional open-ended questions (Table 4) allowed students to provide expanded feedback. Responses were collected anonymously. Demographic information included student age, gender, and stage in medical training. Survey availability was announced during the final class, and a link to Qualtrics was sent via email and placed on the course Canvas page. Two subsequent emails were sent out reminding students of the survey. Students were considered lost to follow up if they did not complete the survey after the second email reminder.

**Table 3:** Likert-style questions and responses

**Impact of Ultrasound Training on Student Confidence in clinical education and practice**		
Please rate how this point of care ultrasound (POCUS) elective has affected your confidence in the following areas:	Response scale: 1 - Much Worse, 6 - Much Better	
Question:	*n*	Mean (±Stddev)
Applying knowledge from the foundations of medicine curriculum to clinical situations	8	5.25 (± 0.89)
Educating patients	8	5.38 (± 0.92)
Succeeding in clinical clerkships and rotations	8	5.63 (± 0.52)
Handling clinical emergencies	8	5.38 (± 0.74)
Passing board exams	8	5.38 (± 0.92)
**Student satisfaction with course**		
Please rate your agreement with the following statements regarding the point of care ultrasound (POCUS) elective:	Response scale: 1 - Strongly Disagree, 6 - Strongly Agree	
WWAMI provided enough ultrasound equipment for the course	8	5.63 (± 0.51)
There were enough instructors to facilitate the scanning practice	8	5.50 (± 0.75)
The POCUS course was integrated effectively in the foundations of medicine curriculum.	8	5.75 (± 0.46)
Faculty from different backgrounds (e.g. Sonographers, Doctors, etc.) brought diverse and valuable perspectives	8	5.75 (± 0.46)
The schedule provided enough class time for me to achieve the learning objectives	8	5.25 (± 1.03)
**Impact of Ultrasound Training on Students’ Confidence in POCUS skills**		
Please rate your agreement with the following statements regarding this elective. After this course, I am confident in my ability to:	Response scale: 1 - Strongly Disagree, 6 - Strongly Agree	
Optimize POCUS images by adjusting settings and presets	8	5.38 (± 0.52)
Choose appropriate POCUS exams based on clinical indications (e.g. chief complaint, vital signs)	8	5.25 (± 0.89)
Acquire optimal POCUS views for clinical interpretation	8	5.38 (± 0.74)
Identify anatomy in each of the instructed views (e.g. PSAx, RUQ, Pelvis, etc.)	8	5.63 (± 0.52)
Identify imaging artifacts in POCUS images	8	5.50 (± 0.76)
**Student experience with teleguidance ultrasound**		
Please rate your agreement with the following statements regarding your point of care ultrasound (POCUS) teleguidance education experience	Response scale: 1 - Strongly Disagree, 6 - Strongly Agree	
The teleguidance/distance teaching I received was effective.	7	5.86 (± 0.38)
I would be interested in more ultrasound training using the teleguidance platform if it were available	8	5.75 (± 0.46)
The teleguidance/distance teaching I received was engaging.	8	5.50 (± 0.76)
The teleguidance/distance teaching I received helped me achieve learning objectives	8	5.63 (± 0.74)
The teleguidance system was easy to use	8	5.38 (± 0.52)

Results from the Likert-style survey questions probing student confidence in future clinical education and practice, course satisfaction, confidence in ultrasound skills, and teleguidance experience. Response scales are indicated with each question

Acronyms: PSAx: parasternal short axis; RUQ: right upper quadrant; WWAMI: Washington, Wyoming, Montana, Alaska, Idaho; POCUS: point of care ultrasound

**Table 4:** Open-ended survey questions and summary responses

Question	Summarized responses
Please share any additional impacts that this course has had on your preparation for medical practice	POCUS is perceived as a valuable clinical skill (*n*=2); interprofessional exposure was valuable (*n*=1).
Please share any additional feedback about the course curriculum and execution (e.g., suggestions for improvements, additions, or highlights from your experience)	Class schedule (day/time) was not ideal (*n*=1)
Please share any additional feedback about your learning outcomes from this course (positive or negative).	Feel more comfortable interpreting images and manipulating the ultrasound probe. (*n*=1)
Please share any additional feedback about the teleguidance component of this course.	No response
What were your motivations for enrolling in the POCUS elective?	To gain exposure to a clinically important skill before clinical education and practice (*n*=7) To reinforce anatomy knowledge (*n*=1)
What was the most valuable part of the POCUS elective?	Guided hands-on practice (*n*=4); exposure to clinicians of various background (*n*=1); greater confidence for clerkships (*n*=1); exposure to POCUS (*n*=1)
What was the least valuable part of the POCUS elective?	Scanning without faculty guidance (*n*=1); course scheduling (day and time) (*n*=1)
What, if anything, would you change to improve the POCUS elective?	Find a better time to hold class (*n*=2); add a musculoskeletal session (*n*=1); more asynchronous videos and greater emphasis on pathology in class (*n*=1)

Open ended questions and condensed responses. *n*= the number of responses containing the summary

Acronyms: *POCUS* Point of care ultrasound.

### Semi-structured interview protocol

Semi-structured interviews were designed with feedback from researchers in the College of Education, Health, and Human Sciences at the University of Idaho with a background in qualitative methodology. Questions probed the domains detailed in the survey above in an open and flexible modality to capture richer insights into students’ experiences. For example, the semi-structured format allows questions to follow topics unforeseen by the researchers during survey design, holding the potential to generate novel feedback. Moreover, verbal communication lets students share a great deal of feedback without the onerous task of producing lengthy written survey responses.

We announced the option to submit interview feedback at the outset of the course and reminded students periodically. Interviews were conducted face-to-face near the conclusion of the course with students who volunteered to participate. Additional informed consent was collected immediately prior to commencing interviews.

### Analysis

Data analyses were performed in Microsoft Excel (Version 2301). Changes in pre-post knowledge tests for all students with matched pre/post scores were assessed with a two tailed T-test. Alpha was set at p ≤ 0.05. Finally, Cohen’s d was calculated to determine the effect size, defined as small (0.2), medium (0.5), large (0.8), or very large (1.3) [56]. Likert responses were reported as mean ± standard deviation (stddev), and open-ended responses were summarized.

Interviews were audio recorded and manually transcribed in Microsoft Word (Version 2301) by JIJ using intelligent verbatim transcription methods [57]. To increase dependability and confirmability [58] of interview findings, notes and comments were embedded in the transcripts to highlight salient passages, recurring responses, or record memos. To promote credibility [58] in the absence of multiple analysts, passages relevant to each survey domain were compiled and triangulated by congruence across the responses of other interviewees and survey data. Representative quotes were selected for the manuscript to promote transferability [58] and bolster student’s survey responses with additional context.

## Results

### Course evaluation

#### Test results

Twenty-four out of 25 students completed the pre-test with a mean score of 18/33 (55%; Highest score = 25/33 (75%); Lowest score = 9/33 (27%); stddev. ± 12.24%), while 19 completed the post-test with a mean score of 26/33 (79%; Highest score = 32/33 (97%); Lowest score = 12/33 (39%); stddev. ± 12.69%). Significant improvement on the overall post-test performance was found (p ≤ 0.0001, Cohen’s d = 1.95).

#### Survey results: demographics

Eight students, all MS1’s, completed the post-course survey. Four (50%) identified as male, three (37.5%) identified as female, and one (12.5%) did not provide a gender. Respondents were between 23 and 29 years of age, with an average age of 25.7 years.

#### Survey results: likert-style questions

Our survey probed four separate constructs relating to their experience in the POCUS elective. Results are shown in Table 3.

#### Survey results: open-ended questions

Open ended survey questions captured several brief responses from participants. These responses generally aligned with the corresponding Likert-style and interview responses. Results are summarized in Table 4.

#### Interviews

Five students (4 Male, 1 Female) submitted interview feedback, supplementing our survey results. When asked about their motivations for enrolling and goals in the course, students expressed their belief that POCUS would become ubiquitous as a valuable and effective tool in medical practice, and that gaining familiarity with the modality would benefit their future as both successful students and providers. One student said:

I can think that there’s an issue, and instead of having to send somebody off, being able to … do an ultrasound on a patient and say "yes or no" this confirms what I thought or – no it’s not what I thought, I need to do something else … [That] is going to save time, it’s going to save pain, and exacerbating of issues for patients, and it’s going to bring a lot of ease of mind [to patients] … Those are all things I value.

We discussed various aspects of students’ course experiences. Mirroring survey findings, several students lauded the interprofessional nature of the course; one student observed the synergism of sonographers, faculty, and physicians and mused: “I think it’s really healthy to have that modelled [for medical students].” In general, students valued the chance to practice a hands-on skill. Moreover, students valued the break in their basic science studies, and the chance to learn a skill that felt directly applicable to their future:

it just feels like a lot of times [our heads] are buried in books … it just doesn’t feel real at all. But there’s something about the ultrasound that … brings you back.

Regarding course resources, students appreciated the succinct video tutorials posted to the course page, brief class lectures, and a focus towards hands-on and guided scanning practice. Moreover, students appreciated the integration of our teaching sessions with basic science topics. However, students were ambivalent towards the question of POCUS as a tool for learning gross anatomy or clinical skills. On one hand, some suggested that learning POCUS was predicated on an *a priori* understanding of the anatomy and evaluation of the relevant system. However, students also relayed the benefits of revisiting content area knowledge and gaining new insight with POCUS:

“I think, truthfully, understanding the anatomy … is a lot different from the ultrasound perspective vs prosection vs books. [for example] … I know the celiac trunk is at T12, but [before using POCUS] I never put together the fact that that’s like, right there [pointing to their body]”

When asked to describe their personal goals for their course participation, every student described a similar desire to master the basics of POCUS and acquire a foundation to build on. All interviewees agreed that they were successful in this pursuit and felt confident in their ability to interpret basic sonograms and capture basic views. However, a common refrain emerged as students acknowledged the need for extensive practice before claiming expertise in sonography: “I thought … oh I’m just going to practice a little bit, but then [realized] I need to practice a lot!”

Students described low initial expectations for the teleguidance teaching modality. However, these reservations were dispelled as students worked with the technology and received an engaging and effective learning experience with some advantages to in person instruction:

I thought the app was going to … feel disconnected. [However, the remote faculty], … was able to give me real-time feedback … [and when compared to an] in person teacher [that’s] tempted to just … [take the probe and acquire the image] like OK, there it is, and that’s cool. But I need to … undergo failure and then find it myself to really have that knowledge constituted [which is the only option when using teleguidance].

Students provided few criticisms of the course. When we asked how we might increase the knowledge and proficiency conferred in future iterations of the elective, students all pointed to a lack of time and bandwidth as a major barrier. One student summed up as follows: “[in medical school] you’re just holding on and there’s just so many different things and you’ve just barely, barely got time.”

## Discussion

### Lessons for rural POCUS education

Complex interventions such as educational initiatives are subject to variable efficacy across unique settings. Therefore, it is important to determine not only what works, but for whom, how, and under what circumstances [59, 60]. In this paper, we described the results of our curriculum development project that investigated the feasibility of developing POCUS teaching within our UME program: the Idaho regional WWAMI campus, located in a rural and medically underserved area of the country.

The use of FOAMed resources was extremely beneficial in three areas of designing and implementing the curriculum: 1) the promotion of intramural expertise through faculty development; 2) reducing the burden on developing novel of didactic content (e.g., figures, video tutorials); and 3) to augment our instruction with clinically sourced sonograms (e.g., the POCUS Atlas). In addition to the use of FOAMed resources, a network of POCUS faculty across UME institutions contributed their insights and experience to our project. These collaborations were facilitated by the ubiquitous access to online communication in email and or teleconferencing software.

Targeting a preclinical student population was beneficial in our case as we were able to lay the groundwork for POCUS competence and proficiency early in medical education, while reducing the clinical focus (e.g., medical decision making) and concomitant need for medical resources and expertise (e.g., clinical faculty, patient care setting, etc.,). Finally, despite the persistent lack of standards in POCUS for UME, literature was available to guide curriculum decisions. However, despite the many successes listed, we face additional challenges and shortcomings to address in the future. For example, implementing formal assessments, including psychomotor evaluations, is paramount to reinforce learning, and better understand students’ proficiency. While many assessment techniques exist for POCUS, they present additional demands on students and institutional resources [61–63].

Moreover, reliance on extramural instruction leaves our program dependent upon volunteers, opening our course to variable availability of instructors and scanning guides. For example, while technology such as teleguidance and videoconferencing platforms make connection to extramural remote faculty possible, lack of access to faculty remains a barrier nationally [7], leading many schools to use volunteers in their POCUS training [18]. Thus, the current national pool of faculty, whether in person or remote, remains insufficient to meet the needs for POCUS training in UME.

To reliably meet the needs of our students, development of more institutional knowledge in POCUS through hiring or faculty development would increase the robustness of our educational training options. Initiatives to include POCUS training in graduate anatomist programs holds potential to help alleviate the shortage of POCUS trained faculty in UME [64, 65]. While these initiatives will take time to bear fruit, we have continued to foster connections to develop POCUS within our local community medical providers. By offering the chance to participate in our POCUS elective to interested local physicians and advanced practice providers we increase the availability of this medical service in our community while also growing the number of local providers to support future students in learning POCUS at our institution.

Finally, while we integrated our POCUS content into the basic science curriculum, the effort remains an optional elective. Our program would benefit from adoption of POCUS teaching standards within the basic science curriculum, either nationally or institutionally, to integrate and expand POCUS content across all regional WWAMI campuses.

### Student outcomes

The success of our curriculum was supported by results of our prospective observational study measuring students’ learning and experiential outcomes. Because online didactic resources are conducive for developing knowledge of POCUS, many researchers point to hands-on skills as the most important and challenging aspect of POCUS courses in UME [10, 12].

Survey (Table 3) and interview results suggest students were satisfied with the availability of equipment and benefitted from interprofessional scanning instructors, including teleguidance instruction used in multiple sessions. Students expressed initial hesitation toward teleguidance, which was quickly dispelled by the utility of the system. Students who used the platform reported that it was effective, engaging, easy to use, and future use of the system would be welcomed, which aligns with prior research [52]. When considered together, remote scanning instructors are a feasible option for increasing the number and availability of instructors in US courses [21, 52, 66]. However, initial skepticism and a national shortage of POCUS faculty busy with their own institutional needs or clinical practice may present challenges to implementation and sustainability of technology focused remote learning experiences in POCUS courses.

Cognitive learning outcomes significantly improved on our written pre/post course test, indicating that students gained knowledge across principles of ultrasound and the various applications we covered (Table 2). Additionally, students felt that we provided sufficient time and resources for them to achieve the learning objectives in the course and reported increased confidence across a range of cognitive domains.

The Society for Academic Emergency Medicine recently outlined various methods for practical assessments of POCUS competency, including Objective Structured Clinical Exams (OSCEs), image review, practical use, and clinical observations [63]. We did not measure psychomotor skill development directly. Nevertheless, students reported increased confidence across all surveyed psychomotor domains of POCUS, including their image acquisition, optimization, and interpretation skills. Moreover, their confidence regarding future clinical performance also increased.

The perceived value of POCUS to physicians was a common motivator for students who enrolled in this elective; a finding in congruence with the broader literature on the perceptions of POCUS in medicine [54, 67]. Social Cognitive Theory positions perceived self-efficacy as a central factor as individuals select their goals and persevere in the face of adversity [68]. Therefore, students’ increased confidence surrounding POCUS and clinical skills may contribute to future success, even in the absence of an objective measure of performance. Still, we grappled with the decision not to include more rigorous assessments in our course and sought further insights from our students.

When interviewing students about the rigor of the elective course, they reported feeling overwhelmed with the basic science curriculum, and expressed hesitancy around adding demanding assessments in the POCUS elective. In addition, no student expressed the goal or expectation to become an expert sonologist during our course. Rather, the most cited objective was to build a foundation in the basics of POCUS. However, the development of more structured assessments may be required as we continue working to expand and formalize POCUS in our basic science curriculum. As a middle ground, participation-based, low-stakes cognitive or psychomotor assessments could elevate student engagement and performance and provide data to evaluate practical competencies without adding excessive pressure to study or prepare [69]. Therefore, the development of formative cognitive exams, OSCEs, or rubrics for clip review and feedback in future iterations of POCUS training may be beneficial [61, 63].

Lastly, the consensus regarding student experience and perception of the course was positive. While participant enjoyment of educational experiences, including satisfaction with the course content, resources, and instructors, is not essential for learning to occur, it is linked with higher quality learning outcomes and improved psychological well-being [53, 69, 70]. Moreover, training in POCUS has been shown to promote engagement, and combat burnout in students and practitioners [68, 70, 71]. In line with these findings, our students reported feeling connected and refreshed by their participation in the elective, particularly the hands-on scanning component. Overall, the results from our students’ experiences and perceptions of POCUS mirror trends reported in the literature [3, 15, 54, 67, 71].

In summary, we developed a POCUS elective in our rural institution in line with the current state of POCUS education in UME. The course was well received, and students valued its hands-on, skills-based nature. Students showed a significant improvement in their knowledge and confidence around POCUS. The bias against basic science in favor of clinical skills among medical students is a longstanding and persistent phenomenon [72], and others have rightly questioned the validity of designing UME curricula by popular demand [54, 67, 73–75]. However, medical students and health care professionals in practice and academics face high rates of depression, burnout, and moral distress [76–80]. Therefore, curating engaging, enjoyable, and valued educational experiences may carry benefits beyond the traditionally measured academic outcomes [69, 71]. Finally, promoting students’ confidence in clinical settings may set them up for continued success [68, 69].

### Limitations

Multiple factors limit the empirical generalizability of our study results: we used a convenience sample of volunteer students at a single medical campus, did not independently validate our surveys or knowledge tests, included a modest sample size, and did not perform extensive qualitative analysis (e.g., thematic analysis) on our interview data. Thus, overall generalizability, such as how teleguidance implementation might work within larger urban settings, larger classes, advanced student populations, unique faculty, or across alternate remote teaching platforms, may not be known from this study. In addition, findings are subject to non-response bias [81], and may not represent the views of all students who enrolled in the course. Finally, we did not evaluate the psychomotor proficiency of our students, relying instead on self-report measures of confidence and an original pre/post knowledge test to inform learning outcomes.

Future research should find ways to increase survey response rates and incorporate competency measures for the psychomotor skills of our students in image acquisition and interpretation. Moreover, we did not assess student performance in other areas of the basic science curriculum that may have been impacted by their elective experience. It would be valuable to determine if students who have completed this elective perform better academically (e.g., basic science curriculum, clerkships), personally (e.g., reduced feelings of burnout, depression, moral injury), or clinically (e.g., changes in clinical practice, POCUS adoption rates).

## Conclusions

We developed a six-month POCUS elective curriculum covering eight content areas in a rural and medically underserved UME setting. Student efforts were instrumental in realizing this elective and garnering necessary resources, and a collaborative and interprofessional approach was invaluable to our success. Results from our knowledge evaluation, course survey, and student interviews suggest the POCUS curriculum was effective in conveying a foundational knowledge of the operation and implementation of POCUS in medical practice. In addition, students reported satisfaction with many aspects of the course including its design, resources, and implementation. More specifically, students pointed to a beneficial integration and application of their basic science knowledge through the course, an increase in confidence regarding their future as students and providers, and the value of a refreshing hands-on experience to break the monotony of the basic science curriculum. Our process adds to the literature in this domain and may provide transferrable insights for similar UME institutions or health professions educators in rural and medically underserved areas.

## Data Availability

The datasets generated and/or analyzed during the current study are not publicly available to protect participant privacy but are available from the corresponding author on reasonable request.

## Abbreviations

POCUS: Point-of-Care Ultrasound
UME: Undergraduate Medical Education
WWAMI: Washington, Wyoming, Alaska, Montana, Idaho
USIG: Ultrasound Interest Group
MS1: First year medical student
MD: Medical Doctor
DO: Doctor of Osteopathy
UWSOM: University of Washington School of Medicine
I-AIM: Indications, Acquisition, Interpretation, Medical decision making
FACP: Fellow of the American College of Physicians
RDMS: Registered Diagnostic Medical Sonographer
IVC: Inferior Vena Cava
eFAST: extended Focused Assessment with Sonography for Trauma
I-TECH: International Training and Education Center for Health
MS: Master of Science
FOAMed: Free Open Access Medical education
MCQ: Multiple Choice Question
Stddev: Standard Deviation
T12: Twelfth thoracic vertebrae
OSCEs: Objective Structured Clinical Exams
